# Glaucoma and dietary links: insights from high-salt intake, the Mediterranean diet, and specific nutrients

**DOI:** 10.3389/fnut.2024.1461748

**Published:** 2024-10-24

**Authors:** Yuqi Yang, Hongyan Zhou, Zhang Hong

**Affiliations:** Department of Ophthalmology, China-Japan Union Hospital of Jilin University, Changchun, China

**Keywords:** glaucoma, nutrients, diet, optic nerve, oxidative stress

## Abstract

Glaucoma, a prevalent and potentially blinding eye disease, is linked to a variety of factors, including elevated intraocular pressure, optic nerve damage, and oxidative stress. In recent years, dietary habits, as a controllable lifestyle factor, have received increasing attention in the prevention and treatment of glaucoma. The purpose of this review was to investigate the effects of dietary factors on glaucoma, with a particular emphasis on two common dietary patterns: the high-salt diet and the Mediterranean diet. In addition, we investigated the association between many particular nutrients (including omega-3 fatty acids, vitamins, caffeine, and minerals) and glaucoma to fully assess the potential involvement of dietary variables in glaucoma pathogenesis, prevention, and treatment. This article reveals the importance of dietary components in glaucoma prevention and explores prospective possibilities for future research by conducting a comprehensive review of previous scientific studies.

## 1 Introduction

Glaucoma, as one of the main causes of visual loss worldwide, is estimated to affect 76 million people aged 40–80 and is expected to increase to 112 million by 2040 (1). The disease threatens and damages the optic nerve (2) and its visual pathways, ultimately leading to permanent vision loss (3, 4), severely affecting the quality of life. Clinically, glaucoma is classified into three main categories: primary, secondary, and congenital. According to the state of the anterior chamber angle, it is further subdivided into closed-angle and open-angle types (5, 6). Of these, primary open-angle glaucoma is the most common (7). As a class of irreversible common blinding diseases (8), glaucoma is characterized by optic nerve damage and visual field loss, and its pathogenesis is complex, involving multiple factors such as genetics, environment, and lifestyle (9–11). Given the complexity of the glaucoma condition and the severe visual damage it may cause, it is important to study its prevention and treatment. While glaucoma is a degenerative disease of the optic nerve (5), lifestyle and dietary habits have a potential impact on preventing the occurrence of glaucoma and improving the prognosis of glaucoma (12–16), which remains controversial and needs to be further explored in rigorous clinical studies.

In recent years, with the deepening understanding of the pathological mechanism of glaucoma, researchers have begun to pay increasing attention to the role of dietary factors in the pathogenesis of glaucoma. Although the exact cause of glaucoma has not yet been fully revealed, existing research suggests that specific dietary habits, such as high-salt diets and Mediterranean dietary patterns, have been found to be associated with the prevalence of glaucoma (17, 18). In this article, we will analyze these two dietary patterns, which differ significantly in terms of nutrient composition and health benefits, particularly in terms of their impact on antioxidant and anti-inflammatory factors, which are thought to have a potentially positive effect on the management of glaucoma. Although there is a dearth of detailed statistical data on the proportion of patients’ dietary choices, the Mediterranean diet has earned worldwide attention and acceptance as a model of a healthy eating pattern (19–23). Furthermore, taking too much salt has become a major worldwide issue (24, 25). Given the popularity of these two diets among the general public, we believe that a study comparing the Mediterranean diet with a high-salt diet would be important for a deeper understanding of the impact of dietary structure on the risk of glaucoma. In addition, some specific nutrients, including omega-3 fatty acids (26–30), vitamins (27, 31–34), caffeine (12, 35–38), and minerals (39–41), may have an impact on the prevention and treatment of glaucoma through various biological pathways such as oxidative stress (42–46), changes in eye blood flow, and regulation of intraocular pressure (12, 47, 48). Overall, dietary factors have a potential impact on the onset and progression of glaucoma through multiple mechanisms, including antioxidant, anti-inflammatory, blood flow improvement, and intraocular pressure regulation (49). In view of this, future studies need to explore these links in further depth and develop reasonable dietary plans for glaucoma patients. The aim of this article is to explore the effects of high-salt intake and Mediterranean dietary patterns on the occurrence and progression of glaucoma in the form of a narrative review; in addition, we analyze the relationship between several specific nutrients, including omega-3 fatty acids, vitamins, caffeine, and minerals, and glaucoma to comprehensively assess the potential roles of dietary factors in the pathogenesis, prevention, and treatment of glaucoma, as well as to summarize and evaluate the results of the current study. Specifically, we focused on relevant studies published in the past 20 years (2004 to 2024) with a view to informing future research and clinical practice. Through an analysis of the effects of several dietary patterns and several specific nutrients on intraocular pressure, optic nerve protection, and ocular blood flow, it becomes clear how these factors indirectly affect how glaucoma progresses. To provide a new perspective and a scientific foundation for comprehensive glaucoma treatment strategies, this review also examines the potential of dietary interventions as adjuvant therapy for glaucoma and future research objectives.

## 2 Method

### 2.1 Literature search strategies

From the inception of the databases to June 2024, we conducted searches across a number of databases, including PubMed, Web of Science, and other libraries. The majority of articles published between 2004 and 2024 were chosen. Predetermined keyword combinations such as “glaucoma,” “intraocular pressure,” “high-salt diet,” “Mediterranean diet,” “Omega-3 diet,” “Omega-3 diet,” and “Omega-3 diet.,” “Omega-3 fatty acids,” “vitamins,” and “minerals” were used.

### 2.2 Inclusion criteria

We mainly included English-language literature published between 2004 and 2024; studies related to the relationship between dietary factors and glaucoma; and types of included studies included, but were not limited to clinical trials, including randomized controlled trials (RCTs) and non-randomized controlled trials (NRCTs), which provided direct evidence on the effects of interventions. Observational studies include prospective cohort studies, retrospective cohort studies, case–control studies, and cross-sectional studies. These types of studies do not prove causality but are useful in exploring associations.

### 2.3 Exclusion criteria

Study design: We excluded case reports, review articles, conference abstracts, etc. as they usually do not provide sufficient data for systematic analysis. Participants: We excluded studies that included only children or adolescents as glaucoma may present differently in adults and risk factors. Language: This study excluded articles published in non-English-language literature. Publication status: We excluded unpublished data, preprints, or gray literature. Duplicate data: We excluded data from duplicate published studies and retained only the original or most recent version of the study. Irrelevance: We excluded studies that were not relevant to glaucoma or diet, even if they contained data on nutrients.

## 3 Pathogenesis of glaucoma

A variety of essential factors, including elevated intraocular pressure, damage to the optic nerve, and oxidative stress, are all components of the complicated pathomechanism of glaucoma.

### 3.1 Physiological basis of elevated intraocular pressure

Elevated intraocular pressure (IOP) is a major risk factor for the development of glaucoma (50–53). The maintenance of intraocular pressure depends on the dynamic balance of aqueous humor, that is, the generation and discharge of aqueous humor remain consistent (54). The aqueous humor enters the anterior chamber through the ciliary body through the pupil, then enters the Schlemm duct through the trabecular meshwork, and finally exits the eye through the Schlemm duct system through the trabecular meshwork (55, 56). When the drainage system is unable to effectively discharge aqueous humor due to various reasons, such as dysfunction of trabecular meshwork cells, Schlemm tube blockage, and angle stenosis, the accumulation of intraocular fluid leads to an increase in intraocular pressure (55, 57–63).

### 3.2 Molecular mechanisms of optic nerve damage

Optic nerve injury is the core pathological process leading to permanent visual loss in glaucoma (3, 4, 7, 64). Elevated intraocular pressure can directly press on the optic nerve (65) and also affect the blood supply to the optic nerve, leading to ischemia and hypoxia (66). This ischemic environment can activate a series of intracellular cascade reactions, including excitotoxicity, inflammatory responses, glial cell activation, and neuronal apoptosis (67). Moreover, Mackenzie et al. have found that elevated IOP may trigger a series of parallel and interacting pathological processes, including direct axonal damage, weight-bearing tissue failure, and disruption of the microvascular supply (68). These processes lead to a progressive loss of structure and function of the optic ganglion cells, eventually causing optic nerve atrophy and visual field defects. In addition, Harada et al. found that mice lacking the GLAST gene developed spontaneous retinal ganglion cell (RGC) reduction and optic nerve degenerative changes, suggesting that reduced GLAST expression leading to glutamate excitotoxicity in the retina may be a potential pathogenetic mechanism for glaucoma (69).

### 3.3 The association between oxidative stress and glaucoma

Oxidative stress is a disruption of the oxidative and antioxidant balance in the body, resulting in high levels of reactive oxygen species (ROS) that exceed the scavenging capacity of the antioxidant defense system, thus causing damage to cellular structure and function (70–74). Oxidative stress plays an important role in the pathogenesis of glaucoma (75–79). In addition, Ferreira et al. found high levels of oxidative stress markers in the atrial fluid of glaucoma patients, suggesting that oxidative stress is involved in optic nerve damage (80). Oxidative stress can cause DNA damage, lipid peroxidation, protein oxidation, and mitochondrial dysfunction (81), all of which are associated with apoptosis in retinal ganglion cells. In addition, oxidative stress promotes extracellular matrix remodeling, exacerbates pathological changes in the trabecular mesh, and further affects atrial water outflow, creating a vicious cycle (79).

Through a variety of intricate processes, dietary antioxidants help to reduce oxidative stress and so decrease the evolution of glaucoma. Direct scavenging of free radicals, suppression of oxidative processes, repair of oxidative damage, upregulation of antioxidant enzyme production, and stabilization of cell membranes are a few examples of these strategies (82–85). For example, vitamin C, as a highly effective water-soluble antioxidant, can directly neutralize free radicals in the aqueous phase, such as superoxide anion and hydroxyl radical, and effectively protect cell membranes and internal components from oxidative damage (82, 86, 87). Vitamin E is a fat-soluble antioxidant that is especially effective at scavenging free radicals produced during lipid peroxidation. This helps to preserve the lipid bilayer of cell membranes’ structural integrity and repairs damage caused by lipid peroxidation to cell membranes, allowing them to resume their regular functions (83, 84). Selenium is one of the key components of glutathione peroxidase, an enzyme that catalyzes the reduction of hydrogen peroxide and organic peroxides to water and the corresponding alcohols by reduced glutathione, significantly reducing the production of free radicals (85, 88). In conclusion, antioxidants play an important role in preventing and mitigating glaucoma, effectively reducing the negative effects of oxidative stress on eye health through a combination of these mechanisms.

## 4 Two dietary patterns

### 4.1 High-salt diet

Existing studies suggest that a high-salt diet may be indirectly associated with glaucoma by affecting blood pressure and other physiological mechanisms (17). A low-sodium diet is a protective factor for hypertension, and excessive salt intake is known to be one of the risk factors for hypertension (89–92). In addition, a high-salt diet may affect intraocular pressure through a series of complex biological mechanisms, and there are findings to support the possibility that increased blood pressure may cause increased intraocular pressure (93), whereas the generation of aqueous humor involves multiple pathways, including secretion, ultrafiltration, and diffusion (94, 95). Among other things, the ultrafiltration process is influenced by plasma colloid osmolality. However, further studies are needed to clarify whether a high-salt diet disrupts the dynamic balance of intraocular fluid by causing systemic fluid retention, which, in turn, affects intraocular pressure.

In addition to being associated with the risk of developing glaucoma, a high-salt diet may also affect the progression of glaucoma. Increased salt intake leads to higher sodium concentrations, leading to stiffening of endothelial cells and reduced release of nitric oxide from vascular endothelial cells (96). Some studies have shown that nitric oxide is a vasodilator that has a positive effect on regulating blood flow and cell viability in the eye and protects vascular endothelial cells and nerve cells. Endothelial cell dysfunction may reduce nitric oxide bioavailability and increase reactive oxygen species production, leading to impaired ocular hemodynamics (97), whereas reactive oxygen species can induce cellular senescence and apoptosis (98), which may ultimately lead to optic nerve cell apoptosis, exacerbating optic nerve damage and further exacerbating the progression of glaucoma. Recently, a researcher conducted an in-depth analysis of the relationship between urinary sodium excretion and glaucoma and its associated characteristics with the help of data from the UK Biobank. The findings suggest that urinary sodium excretion, a biological indicator of dietary sodium intake, may be a key and modifiable risk factor for the development of glaucoma, especially in those individuals with a higher genetic susceptibility. This finding is particularly noteworthy because it not only emphasizes the role of lifestyle factors in the pathogenesis of glaucoma but also points to a potential point of intervention to prevent or delay disease progression by modifying dietary sodium intake (99). In view of the possible influence between a high-salt diet and the onset and progression of glaucoma, a low-salt diet with a reduced intake of processed and fast foods is recommended in clinical practice for glaucoma patients to control intraocular pressure, slow down progression, and improve the quality of life.

### 4.2 Mediterranean diet

The Mediterranean diet, a traditional dietary pattern originating in the countries bordering the Mediterranean Sea, is widely regarded as one of the healthiest diets in the world due to its abundance of vegetables, fruits, whole grains, legumes, nuts, olive oil, and moderate amounts of fish and wine (100–105). The Mediterranean diet emphasizes a whole-food, low-sugar, low-processed food intake with an abundance of vegetables and fruits that are natural antioxidants (106), containing high levels of vitamin C, vitamin E, beta-carotene, and polyphenolic compounds such as flavonoids and anthocyanins (107). These antioxidants play a role in the body in fighting free radicals and mitigating oxidative stress, which is a key factor in the development of glaucoma because it can exacerbate retinal ganglion cell damage and affect optic nerve function (108). Moreno-Montañés et al. find Mediterranean lifestyle as a protective factor for glaucoma incidence as measured by the SUN Healthy Lifestyle Score (SHLS) (109). Thus, the richness of antioxidants in the Mediterranean diet may help to protect the optic nerve against oxidative damage and thus play a positive role in slowing down the process of glaucoma.

## 5 Nutrients and glaucoma

**Figure 1:**
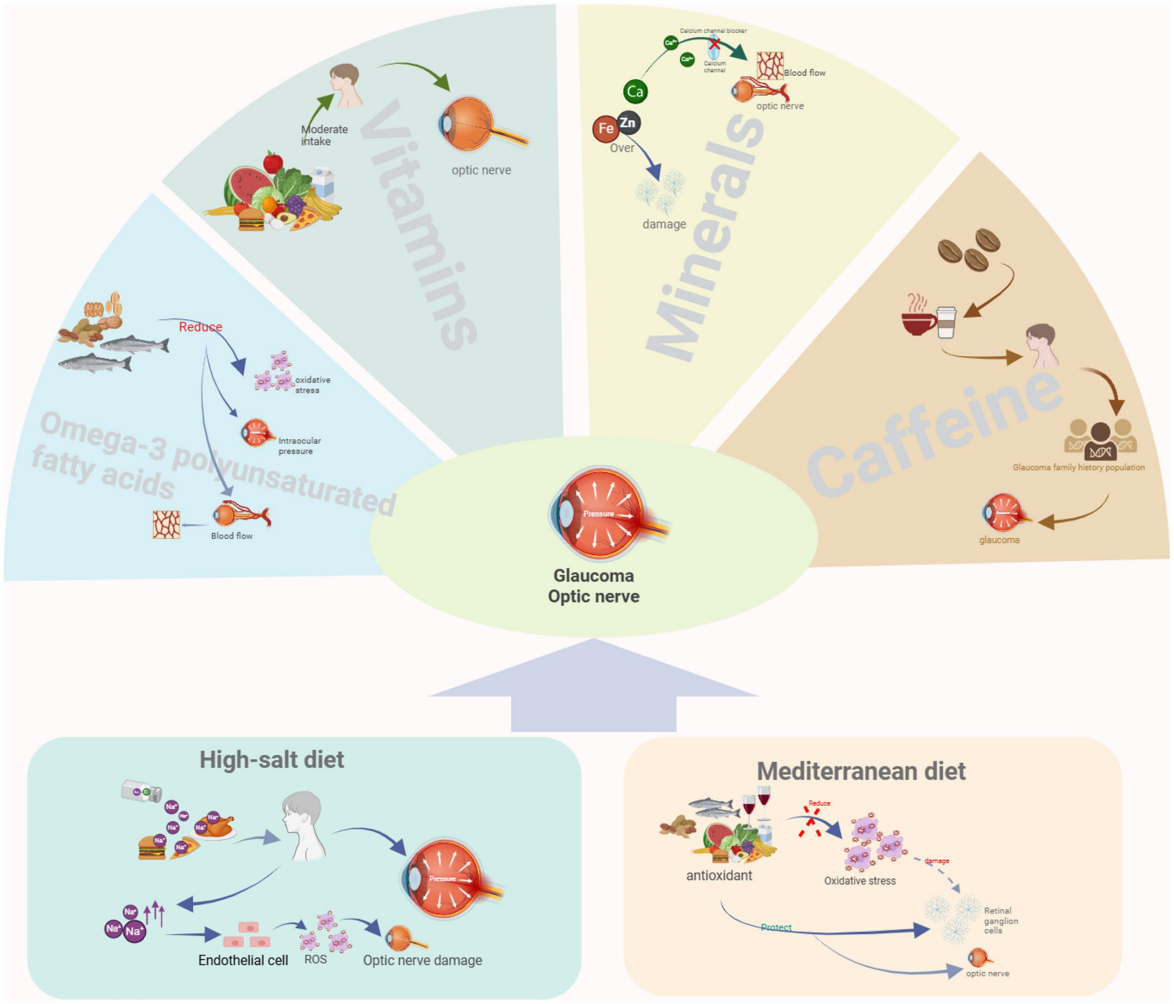
Relationship between two dietary patterns and nutrients with glaucoma. Created with BioRender.com.

Nutrient intake is recognized as a potential factor influencing glaucoma onset and progression (76, 110). The effect of nutrients on glaucoma is a complex, multifactorial, and multi-mechanism process. Although glaucoma is mainly associated with increased intraocular pressure and optic nerve damage rather than being directly caused by nutritional deficiencies, the intake of specific nutrients may help prevent the deterioration of glaucoma or reduce the risk of developing the disease. In the following section, we will analyze the effects of some specific nutrients on glaucoma (Figure 1).

### 5.1 Omega-3 polyunsaturated fatty acids

Omega-3 polyunsaturated fatty acids (PUFA) are a group of essential nutrients whose family members include alpha-linolenic acid (ALA), stearic acid (SDA), eicosapentaenoic acid (EPA), docosapentaenoic acid (DPA), and docosahexaenoic acid (DHA) (111–114). Rich sources of omega-3 in food include deep-sea fish (115–117), such as salmon and mackerel, and plant-based options, such as flaxseeds (118, 119) and walnuts (120, 121), which are all ideal for boosting omega-3 intake in the daily diet.

A cross-sectional study using data from the National Health and Nutrition Examination Survey (NHANES) database (2005–2008) involving 3,865 participants aged 40 years or older found that increasing the proportion of dietary omega-3 fatty acid intake levels in the daily diet may have a protective effect against glaucoma (122). In recent years, studies have shown that a higher intake of omega-3 fatty acids has the potential to reduce the risk of glaucoma and that omega-3 fatty acids may have a protective effect on glaucoma through various mechanisms, such as lowering intraocular pressure, regulating blood supply, relieving inflammation, and reducing oxidative stress (26). 1. Lowering intraocular pressure: Nguyen et al. pointed out through a study on rats that omega-3 fatty acids can lower intraocular pressure by increasing atrial aqueous outflow (30). In addition, oral supplementation with omega-3 fatty acids has been shown to significantly improve IOP (123). However, more research is needed to further validate and elucidate the specific mechanisms and effects of omega-3 fatty acids in lowering IOP. 2. Regulates blood flow in the eye: Eicosapentaenoic acid (EPA) and docosahexaenoic acid (DHA) are two important omega-3 fatty acids (114, 124). A study investigating the fatty acid composition of the blood of patients with primary open-angle glaucoma found that the patients’ blood levels of EPA and DHA were reduced. EPA and DHA can positively regulate systemic microcirculation and improve ocular blood flow, as well as reduce optic neuropathy (125). Kalogerou et al. also found omega-3 to be neuroprotective in the retina in a mouse model of hereditary glaucoma (126). 3. Inflammatory response modulation: Omega-3 fatty acids have anti-inflammatory effects (122, 127–130) and may influence glaucoma progression by modulating the inflammatory response (75).

In conclusion, omega-3 fatty acids, as a potential protective factor, may influence the pathogenesis of glaucoma through multiple pathways. However, the relationship between omega-3 fatty acids and glaucoma still needs to be validated by more longitudinal clinical studies and randomized controlled trials. Therefore, future studies need to further explore the specific roles and mechanisms of omega-3 fatty acids in glaucoma prevention and treatment.

### 5.2 Vitamins (A, E, C, B, K1, and D)

Vitamins, as key dietary supplements, have been widely explored in recent years in studies examining their impact on the common disease of glaucoma (131–137). Several studies have attempted to reveal the potential association of vitamins A, E, C, B, K1, and D with the incidence and severity of glaucoma.

Wang et al. conducted a cross-sectional study in 2013 and found that both supplemental vitamin A and E intake and their serum levels failed to show a correlation with glaucoma incidence. Although the study revealed that low-to-high levels of vitamin C supplementation were associated with a reduced risk of glaucoma, no direct association was found between serum vitamin C concentrations and the prevalence of glaucoma (138). In 2012, Giaconi et al. focused their study on a specific population, older African American women, suggesting that increased intake of fruits and vegetables rich in vitamins A and C and carotenoids may reduce the prevalence of glaucoma in older African American women. However, further randomized controlled trials need to be designed to confirm whether these specific nutrients are effective in modifying the risk of glaucoma (139). Recently, Yang et al. examined the relationship between vitamin B and glaucoma in the US population and found that increased intake of vitamin B6 was negatively associated with the risk of glaucoma (14), providing new evidence for the potential role of vitamin B6 in glaucoma prevention. In addition, an animal model study by Williams et al. revealed the positive effects of vitamin B3 (niacin) on glaucoma and other age-related neurodegenerative diseases, highlighting its potential value in neuroprotection (137). Moreover, the study by Li et al. highlighted the protective effects of vitamin C on the nervous system and suggested that vitamin C may have therapeutic potential for neurodegenerative diseases such as glaucoma (132). Goncalves et al. explored the relationship between vitamin D and primary open-angle glaucoma (POAG) in an elderly population through a case–control approach in their 2015 study, which showed that vitamin D insufficiency was significantly associated with POAG (OR = 2.09, *p* = 0.034), while there was no significant correlation with the severity of the disease. This study used serum 25OHD concentrations as an indicator of vitamin D levels, and although it did not reveal a specific link between vitamin D deficiency and the severity of POAG, it highlights the importance of vitamin D in POAG prevention (140). In addition, a recent study examining the effects of dietary vitamin K supplementation in a rat model of glaucoma revealed an inhibitory effect of high-dose vitamin K1 (VitK1) intake on retinal ganglion cell loss during glaucomatous injury. The researchers concluded that this protective effect may be related to VitK1’s increased expression of stromal GLA protein. Moreover, rats in the high-VitK1 group also exhibited a transient decrease in intraocular pressure (IOP), suggesting that VitK1 may have a potential regulatory effect on the flow of atrial fluid. Overall, the protective effect of VitK1 on retinal ganglion cells may be realized through two mechanisms: first, by lowering the intraocular pressure, which reduces the pressure on the optic nerve, and second, by directly counteracting glaucoma-induced cellular damage through its own neuroprotective effect. Together, these two mechanisms help to minimize the loss of retinal ganglion cells, thereby slowing the progression of glaucoma (141).

When considered collectively, these results reveal that although vitamins as components of dietary supplements have the ability to affect the risk of glaucoma-related diseases, further study is necessary to determine the precise effects of vitamins on the condition. Large-scale, long-term randomized controlled trials should be the primary objective of future research to precisely evaluate the intervention effects of particular vitamin supplements on glaucoma risk in various populations. Deepening our understanding of this complicated disease will require researching the relationships between vitamins and other lifestyle factors, such as dietary practices, physical activity, and smoking status, and how these factors interact to influence the course of glaucoma. Furthermore, given the importance of individual genetic variations, future studies might focus on the relationship between genes and nutrition to promote more individualized and precise glaucoma prevention and treatment strategies.

### 5.3 Minerals (calcium, selenium, zinc, and iron)

Multiple studies reveal a complex link between mineral intake and glaucoma risk (41, 142–148). Calcium is an important mineral for maintaining normal intraocular pressure, and calcium channel blockers are commonly used in glaucoma treatment (142, 143, 149). According to a cross-sectional study, excessive intake of the oxidants calcium and iron may be associated with an increased risk of glaucoma, especially when intake exceeds a certain threshold. Further prospective longitudinal studies are needed to assess whether oxidant intake is a risk factor for glaucoma development and progression (149). Meanwhile, Kastner et al. explored the relationship between calcium channel blocker (CCB) use and the risk of glaucoma in a UK Biobank and showed that the use of CCBs (but not other antihypertensive medications) was associated with an increased chance of glaucoma (odds ratio [OR], 1.39 [95% CI, 1.14–1.69]; *p* = 0.001) (39). In addition, a study by Araie et al. demonstrated that calcium channel blockers (CCBs) improved ocular perfusion and exerted neuroprotective effects on retinal ganglion cells and these CCBs were effective in dilating ocular blood vessels, which facilitated an increase in ocular blood flow. *In vitro* studies also revealed a neuroprotective effect on neurons. This protective effect was also clearly documented in the retinal ganglion cells and photoreceptors of experimental animals (143). Regarding selenium, in a case–control study comparing plasma and atrial selenium levels in patients with primary open-angle glaucoma with those in non-patients, Bruhn et al. found that the odds of glaucoma in the highest tertile of plasma selenium (OR = 11.3; *p* = 0.03) and in the middle tertile of atrial selenium (OR = 0.06; *p* = 0.02) were significantly associated with glaucoma, after adjusting for common glaucoma risk factors (146). In light of the observed increased incidence of glaucoma in some populations taking selenium supplements, Conley et al. explored the mechanism of selenium-induced changes in human trabecular meshwork (HTM) cell homeostasis. Their study revealed that selenium-induced changes in MMP-2 (matrix metalloproteinase-2) and TIMP-1 (matrix inhibitor of metalloproteinase-1) secretion may alter the balance of extracellular matrix transitions in the conventional efflux pathway and lead to elevated intraocular pressure, ultimately leading to glaucoma (147). In addition, it has been found that excessive accumulation of iron and zinc ions causes the loss of retinal ganglion cells (RGCs) and that mitochondrial dysfunction caused by excessive accumulation of iron or zinc, including defects in mitochondrial biogenesis and fusion, as well as processes such as fission and an increase in mitochondrial autophagy, constitutes a potential mechanism of glaucoma causation, accelerating the loss of RGCs (41).

Overall, appropriate intake of minerals is essential for maintaining eye health and preventing glaucoma, but excessive or inappropriate use may be counterproductive. Future studies should continue to explore in depth the optimal range of intake of these minerals, their mechanisms of action, and their interactions with other factors to provide a scientific basis for the development of personalized glaucoma prevention and treatment strategies. In the meantime, clinical practice should take into account individual differences, and mineral supplementation and medication regimens should be monitored and adjusted to achieve optimal therapeutic outcomes and minimize potential risks.

### 5.4 Caffeine consumption

Caffeine, as a central nervous system stimulant, is widely found in coffee, tea, cocoa products, and some drugs (150–154). The results of a series of studies have shown that caffeine at routine intake levels appears to have little effect on IOP and glaucoma risk in the general population. However, higher doses of caffeine intake may be associated with increased intraocular pressure and an increased risk of glaucoma in specific groups, particularly in individuals who are genetically predisposed to high IOP or who have a family history of glaucoma (12, 38, 155). Using statistical modeling in a cross-sectional study from the UK Biobank, Kim et al. revealed that habitual caffeine intake was weakly associated with lower IOP, and the association between caffeine intake and glaucoma was nil. However, among participants with the strongest genetic susceptibility to elevated IOP, greater caffeine intake was associated with higher IOP and a higher prevalence of glaucoma (12). Another prospective cohort study examined the potential association between caffeine intake and the risk of primary open-angle glaucoma (POAG) and primarily found that total caffeine intake was not significantly associated with an increased risk of POAG. However, secondary analyses suggested that caffeine may increase the risk of POAG in individuals with a family history of glaucoma (38). Notably, some association was observed between exfoliative glaucoma and groups with higher coffee consumption. The association between caffeine and caffeinated beverage consumption and the risk of exfoliation glaucoma or exfoliation glaucoma suspected (EG/EGS) was highlighted in a study by Pasquale et al. In this large prospective study, there was a positive correlation between higher coffee consumption and the risk of EG/EGS (156). In addition, Li et al. provided insight into the effects of caffeine on IOP through a systematic evaluation and meta-analysis. The pooled evidence reveals that the effect of caffeine on IOP varies among individuals: In healthy populations, caffeine intake does not cause changes in IOP; in contrast, caffeine intake in patients with glaucoma or ocular hypertension (OHT) tends to increase the IOP significantly (157).

Therefore, although moderate consumption of caffeinated beverages is likely to have little effect on most people, glaucoma patients and high-risk groups should consider moderately limiting their caffeine intake in light of current research findings. Overall, more research is needed to elucidate the relationship between caffeine and glaucoma, but individuals should consider their own conditions and adjust their lifestyle habits appropriately to maintain eye health.

## 6 Conclusion and outlook

Overall, the complex and multifaceted pathomechanisms of glaucoma, a complex degenerative disease of the optic nerve, call for a comprehensive approach to preventive and therapeutic strategies. Dietary factors, as controllable lifestyle components, show potential value in the prevention and treatment of glaucoma. A high-salt diet may exacerbate glaucoma progression through mechanisms that affect blood pressure, ocular blood flow, and oxidative stress, suggesting the importance of a low-salt diet for glaucoma patients. In contrast, the Mediterranean dietary pattern, which is rich in antioxidants, omega-3 fatty acids, and a variety of nutrients beneficial to the optic nerve, may have a protective effect against glaucoma, emphasizing the role of a balanced diet in disease management. Specific nutrients, such as omega-3 fatty acids, demonstrate protective potential against glaucoma through various mechanisms such as lowering intraocular pressure, improving blood circulation, and having anti-inflammatory effects. Research on the correlation between intake of vitamins A, C, E, B-complex, and D, and minerals such as calcium, selenium, zinc, and iron, and glaucoma risk has yielded varying results but generally points to the possible benefits of these nutrients in regulating IOP, antioxidant activity, promotion of blood flow to the eye, and neuroprotection. Vitamins B6 and B3 (niacin), as well as vitamins C and D in particular, have shown potential preventive value against glaucoma. However, excessive intake of minerals such as calcium and iron may be associated with an increased risk of glaucoma, while selenium intake needs to be considered with caution. In addition, caffeine intake and its association with glaucoma risk remind us that even commonly available daily dietary components need to be considered carefully based on individual differences and disease status. Although moderate intake has little effect on the general population, individuals who are genetically predisposed or have a preexisting tendency to glaucoma may be at increased IOP and risk of developing the disease, suggesting that caffeine restriction should be considered in this population.

In summary, increasing the intake of antioxidants and beneficial fatty acids and limiting the excessive intake of potentially harmful minerals and caffeine through rational dietary modification may be important for the prevention and management of glaucoma. However, most of these findings are based on observational and cross-sectional studies, and more high-quality prospective studies and randomized controlled trials are needed in the future to further confirm the causal relationship between dietary factors and glaucoma, as well as to provide more precise dietary guidance to patients. Although the current findings suggest a positive association between dietary factors and glaucoma, the direct causality of the association between these nutrients and glaucoma, as well as their specific roles in different types and stages of glaucoma, remains to be confirmed by more high-quality clinical studies. Future studies should focus on more precisely quantifying the effects of different dietary factors on glaucoma progression, exploring the mechanisms underlying the differential responses between individuals, and conducting long-term interventional studies to develop more personalized and precise dietary guidance protocols. Meanwhile, using modern biomarker technology and big data analysis, the complex interactions between diet and glaucoma can be analyzed in greater depth, providing more scientific and personalized dietary strategies for the prevention and treatment of glaucoma so as to effectively control the disease progression and improve the quality of life of patients.
